# Paediatric Intracranial Aneurysms: Long-term Angiographic and Clinical Outcomes in a Contemporary Series

**DOI:** 10.3389/fneur.2022.684093

**Published:** 2022-03-11

**Authors:** Junlin Lu, Mingtao Li, Yuanli Zhao, Yang Zhao, Xiaolin Chen, Jizong Zhao

**Affiliations:** ^1^Department of Neurosurgery, Beijing Tiantan Hospital, Capital Medical University, Beijing, China; ^2^Department of Neurosurgery, Beijing Electric Power Hospital of Capital Medical University, Beijing, China; ^3^China National Clinical Research Center for Neurological Diseases, Beijing, China; ^4^Stroke Center, Beijing Institute for Brain Disorders, Beijing, China; ^5^Beijing Key Laboratory of Translational Medicine for Cerebrovascular Disease, Beijing, China; ^6^Beijing Translational Engineering Enter for 3D Printer in Clinical Neuroscience, Beijing, China; ^7^Department of Neurosurgery, Peking University International Hospital, Peking University, Beijing, China

**Keywords:** intracranial aneurysm, microsurgery, endovascular therapy, paediatric, outcomes

## Abstract

**Objective:**

Paediatric aneurysms are rare and difficult to treat. Studies on the long-term angiographic and clinical outcomes conducted within the past decade are lacking. We aimed to investigate the clinical and radiographic outcomes of paediatric aneurysms treated with different strategies in a contemporary series.

**Methods:**

We performed a retrospective medical record review of paediatric patients examined at our institution between 2011 and 2018. Patient charts were retrospectively reviewed for age, presentation, type and location of the aneurysm, modalities of treatment, complications, and clinical and angiographic outcomes. The rates of aneurysm recurrence and *de novo* formation were determined.

**Results:**

We evaluated 61 patients (mean age, 11.6 years; 23 females, 38 males) with 69 intracranial aneurysms. Their presentations included headache, neurological deficits, aneurysmal subarachnoid haemorrhage, incidental aneurysm, and traumatic subarachnoid haemorrhage. Of the aneurysms, 30 (49.2%) were giant. Forty-five (73.8%) patients underwent treatment for their aneurysms, and 16 (26.2%) patients were managed conservatively. The perioperative morbidity rate was 17.8%. There were no deaths. The long-term morbidity rate was 4.6%. The clinical outcomes were favourable in 82.2 and 95.3% at discharge and follow-up, respectively (mean, 41.5 months; range, 1.5–9 years). For treated aneurysms, 2/43 (4.6%) risk of aneurysm recurrence, 1/43 (2.3%) risk of aneurysm bleeding, 1/43 (2.3%) risk of *de novo* aneurysm formation. The annual bleeding, recurrence, and *de novo* formation or growth risk were 0.7, 1.4, and 0.7%, respectively.

**Conclusions:**

In neurovascular centres where microsurgical and endovascular options are available, most children with intracranial aneurysms can be successfully treated with low morbidity and mortality. However, they have higher rates of recurrence and a greater risk of *de novo* formation or growth than their adult counterparts, which mandates lifelong follow-up.

## Introduction

Paediatric aneurysms are rare and account for approximately 1–5% of intracranial aneurysms (1, 2). Aneurysms in paediatric patients differ significantly from their adult counterparts in several aspects (3). Currently, two large institutions have shared their experiences in treating paediatric aneurysms over more than 20 years (4). However, the past two decades have witnessed a burgeoning enthusiasm for endovascular treatment and a gradual shift from traditional surgical approaches at some centres (5, 6). The development of the operative technique may lead to heterogeneity in outcomes during such a long period with the explosive technological progress. Rational decision-making for paediatric aneurysms requires a comparison of the safety and efficacies of the newer endovascular therapies with those reported in the literature. With the rapid development, endovascular therapies have gradually become effective and safe for treating aneurysms. The progress of endovascular therapies has also facilitated the development of hybrid therapies and further improvement in the effectiveness of treatment for aneurysms. Consequently, the results from older publications may not apply to the current practise environment. This study aims to provide data on this rare condition based on the experiences of clinicians in a single centre within the recent decade.

## Materials and Methods

### Patient Population

The study protocol was approved by the ethics committee of our institution. Written informed consent was obtained from all participants or their guardians on admission.

Between 2011 and 2018, 61 paediatric patients (<18 years) with 69 intracranial aneurysms were treated at our institution, and their medical records were retrospectively reviewed. The patients were evaluated on admission by neurosurgeons and interventional neuroradiologists. Follow-up information was obtained by reviewing the most recent hospital records and using telephone questionnaires.

### Diagnostic Imaging

Pre- and Post-treatment CT and/or digital subtraction angiography (DSA) data were obtained, and the dictated reports were reviewed. The perioperative period encompassed the hospitalisation period until 1 month after discharge. The aneurysms were classified as fusiform/dissecting, infectious/mycotic, traumatic/iatrogenic, or saccular (1). The aneurysms with sizes of >2.5 cm were categorised as giant (7). Aneurysm recurrence was defined as the regrowth of a previously treated aneurysm. *De novo* aneurysm formation was defined as the formation of a new aneurysm or growth of an existing aneurysm at a location remote from the treated aneurysm. The annual percentage rates were determined by dividing the number of clinical events (recurrence or *de novo* formation) by the number of patient-years of observation for the population (8).

### Treatment

A multidisciplinary team which included cerebrovascular neurosurgeons and interventional neuroradiologists made management decisions for individual patients. After the angiographic diagnosis, the patients were treated by microsurgery, endovascular intervention, a combination of the two, or observation. The microsurgical treatment consisted of direct neck clipping, parent artery reconstruction, or trapping with/without bypass performed by cerebrovascular neurosurgeons. The endovascular treatment by the interventional neuroradiologists involved the use of traditional stents, coiling, or pipeline embolisation devices (PED; Medtronic, Minneapolis, Minnesota, USA) delivered through microcatheters after transfemoral super-selective catheterisation of the involved intracranial arteries. Alternatively, for aneurysms not amenable to coil obliteration with preservation of the parent artery, a parent artery occlusion via detachable balloons or coils was performed after the demonstration of adequate collateral circulation by the awake balloon occlusion test. If a significant aneurysm neck remnant was detected on follow-up surveillance angiography, retreatment was performed with further endovascular treatment, microsurgery, or observation. Combined approaches were used when one treatment technique could not achieve ideal results. The cases of aneurysms with high risks of morbidity and mortality associated with endovascular and microsurgical procedures or those of patients who were unwilling to undergo interventional procedures were observed.

### Follow-Up

The microsurgical patients were clinically followed in the neurosurgical outpatient clinic 3 and 6 months after surgery. Patients in the endovascular group were followed up using DSA and clinical examinations for 3–6 months and 6–12 months after the initial treatment. Follow-up DSA or CT angiography was performed annually to evaluate aneurysmal stability and the growth of the patients in the untreated observation group. In addition, imaging examinations were performed once when there were new symptoms. The neurologic condition of the patient was evaluated using the modified Rankin Scale (mRS) score. The preoperative neurologic condition was used as a reference point, and the clinical outcomes were expressed as the difference between the last follow-up and baseline mRS scores (<0, improved; =0, unchanged; >0, worse; or dead). Thus, asymptomatic patients who persistently had no neurologic deficits were classified as unchanged.

### Data Analysis

All analyses were conducted using IBM SPSS statistical software 26.0 (IBM Corp., Armonk, NY, USA). Statistical significance was set at *p* < 0.05 for a 95% confidence interval (CI). The original baseline differences between the ruptured and unruptured groups were evaluated using a *t*-test for continuous variables and the Chi-squared test for categorical variables. Given the potential confounding effects of selection and routine biases, we compared the characteristics in the present study with those of other published medical literature.

## Results

### Baseline Characteristics

A summary of the characteristics of the paediatric patients with aneurysms treated at our institution is presented in Table 1. The participants included 23 females and 38 males aged 2–17 years (mean: 12 ± 5 years). The female-to-male ratio was 1:1.7. Neurological deficits were present in 16 patients (26.2%). Twenty-four patients (39.3%) presented with headaches. Subarachnoid haemorrhage (SAH) was related to an aneurysm in 12 patients (19.7%) and trauma in one patient (1.7%). Aneurysms were incidentally discovered in 8 patients (13.1%). Six patients (9.8%) had multiple aneurysms. There were 69 aneurysms in total (Table 2) and 43% of them measured >2.5 cm. Fifty-six saccular aneurysms were observed in 48 patients. Twelve fusiform aneurysms were observed in 12 patients. A traumatic aneurysm was observed in one patient. None of the patients had a family history of aneurysms or other genetic diseases associated with aneurysms.

**Table 1:** Characteristics of patients.

**Characteristic**	**Total**	**Ruptured**	**Unruptured**	***P*-value**
	**(*n* = 61)**	**(*n* = 13)**	**(*n* = 48)**	
Age, years	11.6 ± 4.5	11.2 ± 5.1	11.8 ± 4.4	0.667
**Sex**				0.061
Female	23 (37.7)	2 (15.4)	21 (43.8)	
Male	38 (62.3)	11 (84.6)	27 (56.3)	
mRS score on admission	1.4 ± 1.1	2.3 ± 1.8	1.2 ± 0.7	<0.001
**Presenting symptoms**				<0.001
Headache	24 (39.3)	0 (0)	24 (50.0)	
Neurological deficits	16 (26.2)	0 (0)	16 (33.3)	
SAH	12 (19.7)	12 (92.3)	0 (0)	
Incidental	8 (13.1)	0 (0)	8 (16.7)	
Traumatic	1 (1.7)	1 (7.7)	0 (0)	
**Morphology**				0.026
Saccular	48 (78.7)	12 (92.3)	36 (75.0)	
Fusiform/dissecting	12 (19.7)	0 (0)	12 (25.0)	
Traumatic	1 (1.6)	1 (7.7)	0 (0)	
Multiple	6 (9.8)	2 (15.4)	4 (8.3)	0.449
Giant (>2.5 cm)	30 (49.2)	1 (7.7)	29 (60.4)	0.001
Posterior circulation	22 (36.1)	5 (38.5)	17 (5.4)	0.839
Recurrent aneurysm	5 (8.2)	2 (15.4)	3 (6.3)	0.287
**Treatment modality**				0.006
Microsurgery	20 (32.8)	9 (69.2)	11 (22.9)	
Endovascular	25 (41.0)	3 (23.1)	22 (45.8)	
Conservative	16 (26.2)	1 (7.7)	15 (31.3)	
Poor outcome^*^	2 (3.8)	2 (15.4)	0 (0)	0.012

*Data are presented as n (%) unless otherwise indicated. Mean values are provided with SD*.

* *Fifty-two patients were available at the last follow-up*.

**Table 2:** Location of aneurysms.

		**Endovascular**	**Microsurgery**	**Observation**
	**Total = 69**	**group**	**group**	**group**
		**(*n* = 27)**	**(*n* = 25)**	**(*n* = 17)**
**Anterior circulation**	45 (65.2)	14 (51.9)	21 (84.0)	10 (58.8)
Cavernous ICA	11 (15.9)	7 (25.9)	1 (4.0)	3 (17.6)
Supraclinoid ICA	3 (4.3)	1 (3.7)	0 (0)	2 (11.8)
Ophthalmic	3 (4.3)	1 (3.7)	1 (4.0)	1 (5.9)
Bifurcation	2 (2.9)	1 (3.7)	1 (4.0)	0 (0)
PCoA	2 (2.9)	0 (0)	1 (4.0)	1 (5.9)
ACA	4 (5.8)	2 (7.4)	1 (4.0)	1 (5.9)
ACoA	2 (2.9)	0 (0)	2 (8.0)	0 (0)
Pericallosal	1 (1.5)	0 (0)	1 (4.0)	0 (0)
MCA	17 (24.6)	2 (7.4)	13 (52.0)	2 (11.8)
**Posterior circulation**	24 (34.8)	13 (48.1)	4 (16.0)	7 (41.2)
PCA	5 (7.2)	2 (7.4)	2 (8.0)	1 (5.9)
SCA	1 (1.5)	0 (0)	0 (0)	1 (5.9)
Basilar trunk	7 (10.1)	4 (14.8)	0 (0)	3 (17.6)
Basilar bifurcation	1 (1.5)	0 (0)	0 (0)	1 (5.9)
PICA	2 (2.9)	1 (3.7)	1 (4.0)	0 (0)
Vertebral artery	8 (11.7)	6 (22.3)	1 (4.0)	1 (5.9)

*ICA, internal carotid artery; PCoA, posterior communicating artery; ACA, anterior cerebral artery; ACoA, anterior communicating artery; MCA, middle cerebral artery; PCA, posterior cerebral artery; SCA, superior cerebellar artery; PICA, posterior inferior cerebellar artery*.

### Aneurysm Management

Overall, 45 patients underwent treatment for 52 aneurysms (Table 3). Microsurgery was performed with standard craniotomy based on the location of 25 aneurysms (48.1%). The most common aneurysm site in the microsurgery group was the middle cerebral artery (MCA; 52%). Twenty-one aneurysms (44%) were treated with primary clipping. Two aneurysms (3.8%) were trapped with bypass, and the parent artery was reconstructed for 1 (1.9%). Twenty-seven aneurysms underwent endovascular treatment (51.9%). The cavernous internal carotid and vertebral arteries (VAs) were the most frequent aneurysm sites. They accounted for 25.9 and 22.3%, respectively. Primary coiling was used to treat 9 aneurysms (17.3%). Seven aneurysms (13.5%) were treated with PED. Parent artery occlusion via a detachable coil or balloon was performed for 12 aneurysms (23.1%). Sixteen patients were managed conservatively with close follow-up and serial imaging.

**Table 3:** Management of paediatric aneurysms.

	***N***	**Giant**	**Saccular**	**Posterior**
				**circulation**
**Microsurgery**	25 (48.1)	7 (30.4)	25 (56.8)	4 (23.5)
Direct neck clipping	22 (42.3)	4 (17.4)	22 (50.0)	4 (23.5)
Parent artery reconstruction	1 (1.9)	1 (4.3)	1 (2.3)	0 (0)
Trapping with bypass	2 (3.8)	2 (8.7)	2 (4.5)	0 (0)
**Endovascular treatment**	27 (51.9)	16 (69.6)	19 (43.2)	13 (76.5)
Traditional stent and coiling	8 (15.4)	3 (13.0)	7 (15.9)	2 (11.8)
PED	7 (13.5)	5 (21.7)	6 (13.6)	3 (17.6)
Endovascular trapping	12 (23.1)	8 (34.8)	6 (13.6)	8 (47.1)
Total	52	23	44	17

No deaths were recorded during the perioperative period. Perioperative complications were noted for 3 procedures (6.7%). There were postoperative infarctions with aphasia and hemiplegia in the microsurgery group, two cases of trapping with the bypass procedures, and one primary clipping procedure. There were no postoperative complications.

### Follow-Up

In our series, 45 patients with 52 aneurysms underwent microsurgical or endovascular treatments during separate admissions. They were all discharged from the hospital. Based on the mRS score, 37 cases (82.2%) had good outcomes (mRS scores of <2), and 8 (17.8%) had poor outcomes (mRS scores of ≥ 2) (Table 4). All patients had clinical follow-up lasting for 3–6 months after their original surgery. After excluding 2 patients (4.4%) who followed-up for <1 year, 43 patients (95.6%) were included for the analysis of the outcomes. At the last follow-up (mean, 41.5 months; range, 1.5–9 years), 41 patients (95.3%) had mRS scores of 0 or 1. One patient (1/43, 2.3%) with a residual aneurysm on postoperative angiography experienced SAH 20 months after surgery and underwent another microsurgery to clip the aneurysm at our institution, creating an annual haemorrhage risk of.7% in treated aneurysms. Two cases of recurrence (2/43, 4.6%) and one case of *de novo* formation (1/43, 2.3%) were observed in the other patients during the follow-up, creating an annual recurrence risk of 1.4% and annual *de novo* aneurysm formation or growth risk of.7% in treated aneurysms. The rate of treatment-related morbidity (mRS score of >1) was 17.8% during the perioperative period and 4.7% at the last follow-up. Two patients (4.7%) had neurological deficits (one had bilateral oculomotor nerve paralysis due to a motor vehicle accident and 1 had trouble with fine motor skills). Thirty-five patients (81.4%) had improved neurological function, and the preoperative symptoms were resolved at the last follow-up.

**Table 4:** Perioperative and follow-up outcomes of paediatric aneurysms.

	**Treatment**			**Observation**
	**Total**	**Endovascular**	**Microsurgery**	
	**(*n* = 45)**	**(*n* = 25)**	**(*n* = 20)**	**(*n* = 9)**
**Perioperative outcome**				
Postoperative infarctions	3 (6.7)	0 (0)	3 (15.8)	—
Aneurysm residual	9 (20.0)	9 (36.0)	0 (0)	—
**mRS score at discharge**				
1	37 (82.2)	23 (92.0)	14 (70.0)	8 (88.9)
2	3 (6.7)	1 (4.0)	2 (10.0)	0 (0)
3	2 (4.4)	0 (0)	2 (10.0)	1 (11.1)
4	3 (6.7)	1 (4.0)	2 (10.0)	0 (0)
Follow-up outcome^*^				
Follow-up period, months	41.5	39.0	44.8	38.9
SAH	1 (2.3)	0 (0)	1 (5.3)	0 (0)
Aneurysm recurrence	2 (4.6)	1 (4.2)	1 (5.3)	—
*De novo* aneurysm formation	1 (2.3)	0 (0)	1 (5.3)	0 (0)
Aneurysm enlargement	—	—	—	2 (22.2)
**mRS score at last follow-up**				
0	31 (72.1)	21 (87.5)	10 (52.6)	7 (77.8)
1	10 (23.3)	2 (8.3)	8 (42.1)	2 (22.2)
2	1 (2.3)	0 (0)	1 (5.3)	0 (0)
3	0 (0)	0 (0)	0 (0)	0 (0)
4	1 (2.3)	1 (4.2)	0 (0)	0 (0)
Neurological function improved	35 (81.4)	21 (87.5)	14 (73.7)	7 (77.8)

*SAH, subarachnoid haemorrhage*.

*Data are presented as number (%)*.

* *There were 24 patients in the endovascular group and 19 patients in the microsurgery group at the last follow-up*.

Of the 16 patients placed under conservative observation, 1 was lost to follow-up after the initial evaluation, 6 received treatment at other institutions, and the remaining 9 had an average follow-up duration of 38.9 months (range, 1–7.5 years). These patients underwent serial DSA or CT angiography for aneurysm growth. One patient showed spontaneous occlusion (1/9, 11.1%) of the aneurysm and two patients (2/9, 22.2%) showed a minimal enlargement of the aneurysm. None of the aneurysms ruptured during the follow-up.

### Case Examples

**Figure 1:**
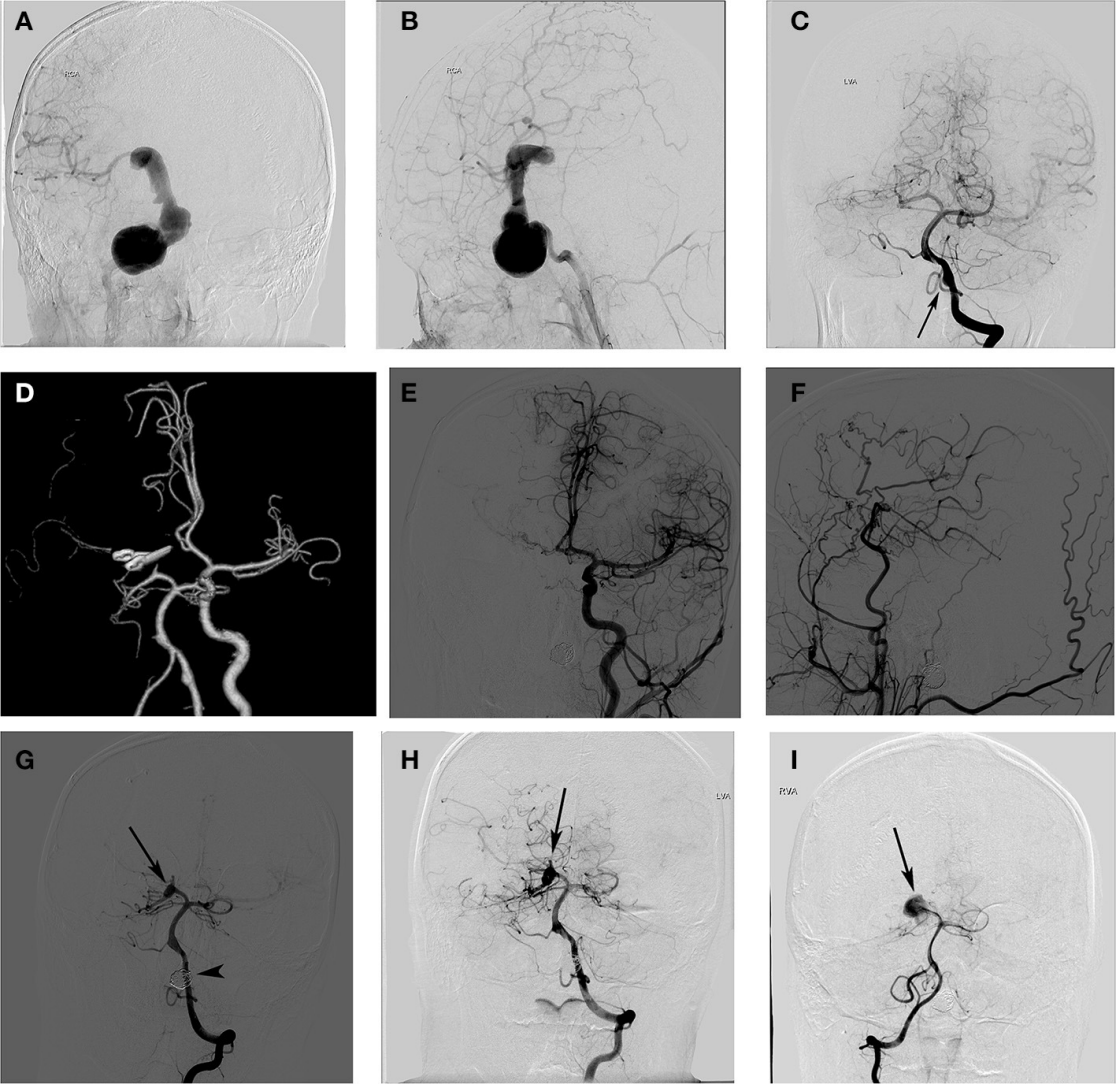
A schoolboy presented with blurred vision and headache. The right internal carotid artery (ICA) angiogram shows a giant ICA aneurysm **(A,B)**, and the left vertebral artery (LVA) angiogram shows a dissecting aneurysm **(C)**. Postoperative CT angiography showed complete obliteration of the aneurysm **(D)**. The angiograms of the left **(E)** and right ICA **(F)** 2 years later showed no recurrent aneurysm and a good filling of the distal middle cerebral artery (MCA) branches via the bypass. Anterior projections of the left VA angiogram **(G)** showed complete occlusion of the left VA aneurysm (arrowhead) and a *de novo* aneurysm formation of the right posterior cerebral artery (PCA) (arrow). A follow-up angiogram of the right PCA showed no aneurysm enlargement 18 months after it was detected **(H)** and a significant aneurysm enlargement 5 years after it was detected **(I)**.

A schoolboy previously diagnosed with aneurysm presented with blurred vision and recurrent headache that had lasted for 8 months. Right internal carotid artery (ICA) angiograms showed a giant aneurysm from the cavernous ICA to the ICA bifurcation (Figures 1A,B). An angiogram of the left VA showed a dissecting aneurysm at the left VA (Figure 1C). We first treated the giant aneurysm with microsurgery. During the surgery, the lesion was categorised as a thrombotic aneurysm. The aneurysm was trapped by ligating the proximal ICA and clipping the MCA bifurcation. A bypass from the superficial temporal artery (STA) to the MCA was performed to prevent thrombotic complications. CT angiography performed 7 days Post-surgery showed complete obliteration of the aneurysm (Figure 1D). He developed an infarction after surgery, which manifested as hemiplegia. After a protracted hospital course, the patient's neurological function improved, and he was discharged with an mRS score of 2.

The patient was regularly followed-up Post-surgery, and DSA confirmed the complete occlusion of the Right ICA giant aneurysm and patency of the STA bypass graft (Figures 1E,F). The enlargement of the left VA (LVA) dissecting aneurysm was observed during the follow-up. Thus, 2 years after the first surgery, the LVA dissecting aneurysm of the patient was treated with coils. Postoperatively, he had no neurological deficits and was discharged from the hospital. Imaging studies 6 months after the endovascular treatment showed complete occlusion of the LVA dissecting aneurysm and a *de novo* aneurysm formation in the right posterior cerebral artery (PCA) (Figure 1G). We suggested that the right PCA *de novo* aneurysm needed treatment to reduce the risk of bleeding, but the patient's guardian refused surgery. Therefore, the right PCA *de novo* aneurysm was managed conservatively. An angiogram at 18 months after detection showed no enlargement of the aneurysm (Figure 1H). The most recent angiogram (5 years after detection) showed a significant increase in the right PCA aneurysms (Figure 1I). The patient was well during his 7-year follow-up visit.

## Discussion

Our study demonstrated that paediatric aneurysms tend to be larger, have more complex shapes, and are more likely to be located in the posterior circulation than adult aneurysms, which is consistent with previous reports (2, 9, 10). Between 2011 and 2018, paediatric aneurysms accounted for 1% of aneurysms treated at our institution. Although there are published series, it is difficult to make rational conclusions and treatment recommendations for the paediatric population due to the small sample size. There have also been discrepant clinical descriptions and reports on treatment outcomes. There have been reports that only 40% of patients have good outcomes (11). In this paper, we report the findings of a single centre related to paediatric aneurysm treatment within the last decade.

We demonstrated a male preponderance (M/F 1.7:1) for aneurysms in our population, as previously reported (12). A previous series suggested that the age at presentation follows a bimodal distribution: birth to 6 years of age and 8 years of age to adolescence (13). However, the majority of our patients (68.9%) did not present with aneurysms until 10 years. In our series, 86.9% of the aneurysms were symptomatic. Our institution is a major referral centre for cerebrovascular disease in China. Therefore, most patients in our cohort had unruptured aneurysms. SAH was a less common presentation in our series compared with previous studies. In addition, aneurysms in patients at our institution tended to be larger and had complex shapes. Giant aneurysms accounted for nearly 50% of the cases in our series. The patterns of referral to the relevant institutions may contribute to the differences between our series and previous reports. The anterior circulation (64%) was the most frequent site of aneurysm formation (posterior circulation: 36%, which was 3-fold higher than the proportion in adults) (7). Generally, giant and fusiform/dissecting aneurysms require treatment because of their poor prognoses if untreated. They have risks for haemorrhagic and ischaemic stroke. Therefore, the high incidence of complex lesions in the paediatric population necessitates effective treatment.

Some studies have reported surgical mortality rates of 1.3–10.4%. Most of the series are small and were conducted several years ago. However, more paediatric patients with intracranial aneurysms can be successfully treated with low morbidity and mortality, given the development of the endovascular technique and multidisciplinary cooperation. The previous data may not reflect the current situation for paediatric aneurysms. The treatment-related morbidity and mortality rates in this study were low for the surgical and endovascular groups than those reported in the medical literature (Table 5). In our contemporary series, the perioperative mortality rate was zero. The outcomes were favourable (mRS scores of <2) in 82.2% of our patients at discharge and 95.4% at the last follow-up.

**Table 5:** Aneurysm characteristics in various case series and meta-analyses.

**Series**	**Current**	**Lasjaunias et al. (1)**	**Hetts at el. (4)**	**Kakarla et al. (14)**	**Mehrotra et al. (2)**
Patients (No.)	61 (69 aneurysms)	59 (75 aneurysms)	77 (103 aneurysms)	48 (72 aneurysms)	57 (73 aneurysms)
**Aetiology/morphology**					
Fusiform	20%	56%	31%	39%	—
Saccular	78%	27%	46%	45%	—
Infectious	0%	14%	12%	7%	—
Traumatic	2%	3%	14%	10%	—
Giant (>2.5 cm)	49%	—	11%	23%	19%
Multiple	10%	15%	16%	31%	19%
Posterior circulation	36%	27%	22%	24%	29%
Age	12 yr (2 yr to 18 yr)	7.6 yr (8 days to 15 yr)	12 yr (3 mo to 18 yr)	12 yr (8 mo to 18 yr)	13 yr (8 mo to 18 yr)
Sex (% male)	62%	59%	48%	80%	46%
Haemorrhage	21%	54%	32%	51%	88%
Morbidity	3.4%	—	12%	14%	14%
Mortality	0%	10.4%	1.3%	3%	9%

In our series, 5 patients with SAH had poor neurological function (mRS score 3–5) on admission, which was resolved at discharge and follow-up. Unlike aneurysmal SAH, there are moderate-to-severe disability and mortality rates in adult patients (15). Furthermore, of the aneurysms managed conservatively, 2 out of 9 (22.2%) showed aneurysm growth in 3.2 years follow-up. Thus, the treatment of aneurysm is essential for paediatric patients. Microsurgery or endovascular treatment may improve the outcomes of paediatric patients with ruptured aneurysms even if they have a relatively poor preoperative neurological function. Therefore, we recommend a positive attitude towards paediatric aneurysms.

Angiographic outcomes are indispensable for assessing the efficacy of microsurgical or endovascular treatment of aneurysms. The aneurysm obliteration rate was 100% in our microsurgical group, while there were 9 residuals for 27 aneurysms treated endovascularly. This rate, however, includes seven aneurysms that were treated with PED, and the dome of the aneurysm was loosely packed by coils which would show as residuals on angiography. We observed two recurrences during the angiographic follow-up of 43 treated aneurysms. The recurrence rate was 4.6%, with a 1.4% annual risk of recurrence. This was 2-times higher than that of adults according to a previous study (7). In addition, it should be noted that 3 patients had recurrent aneurysms that had been previously treated at other institutions. The actual recurrence rate may be higher than 4.6%. There was no significant difference between the recurrence rates in the microsurgical and the endovascular treatment groups (5.3 vs. 4.2%). In our series, none of the recurrences were associated with a residual on immediate postoperative angiography. Some authors have suggested that microsurgery may be more efficacious in eliminating the aneurysm, and its effects are more durable over the extended lifetime of these patients (11). Our results show that both modalities of treatment have similar outcomes. There was one case of *de novo* aneurysm formation after microsurgery in our series. The risks of recurrence and *de novo* formation in children are much higher than those in the adult population, which is a major concern given the life expectancy of children. As reported previously by other investigators, new and enlarging aneurysms were also observed in patients initially treated surgically and endovascularly (16). Moreover, some studies reported that new aneurysms occurred 5 or more years after the initial treatment (4, 14). Therefore, the delayed appearance of *de novo* lesions necessitates the follow-up of these paediatric patients into adulthood.

The study has limitations. There may have been selection bias. Most included patients had unruptured aneurysms, and those with emergencies treated at other institutions were excluded. Therefore, the conclusions may not be generalisable to all paediatric aneurysm patients. In addition, this is a single-centre study spanning ~9 years, and not all patients were regularly followed up. Therefore, the results may have been affected by attrition bias.

## Conclusion

Paediatric aneurysms are uncommon and complex. They often require advanced surgical and endovascular techniques and a skilled multidisciplinary team to achieve good outcomes. There are higher annual risks of recurrence and *de novo* formation in paediatric than in adult patients, along with the potential for new aneurysms during the long-life expectancy of children. We, therefore, advocate lifetime vigilance and angiographic follow-up.

## Data Availability Statement

The data that support the findings of this study are available from the corresponding author upon reasonable request.

## Ethics Statement

The studies involving human participants were reviewed and approved by IRB of Beijing Tiantan Hospital, Capital Medical University. Written informed consent to participate in this study was provided by the participants' legal guardian/next of kin.

## Author Contributions

JL designed the study, wrote the manuscript, researched the data, and contributed to the discussion. ML designed the study, contributed to the discussion, and edited the manuscript. YuZ, XC, and YaZ contributed to the discussion and edited the manuscript. JZ designed the study, researched the data, reviewed and edited the manuscript, and contributed to the discussion. All authors contributed to the article and approved the submitted version.

## Funding

This work was supported by National Key Technology Research and Development Program of the Ministry of Science and Technology of China (2015BAI12B04), Beijing Science and Technology Supporting Plan (D16110000381605), Beijing Municipal Administration of Hospitals' Mission Plan (SML20150501), and the National Natural Science Foundation of China (81571110 and 81771234).

## Conflict of Interest

The authors declare that the research was conducted in the absence of any commercial or financial relationships that could be construed as a potential conflict of interest.

## Publisher's Note

All claims expressed in this article are solely those of the authors and do not necessarily represent those of their affiliated organizations, or those of the publisher, the editors and the reviewers. Any product that may be evaluated in this article, or claim that may be made by its manufacturer, is not guaranteed or endorsed by the publisher.
